# Supplementary material for: Over four minutes of pyruvate T1 using chemically and physically induced deceleration of relaxation
Source: Nat Commun. 2026 May 21;17:4561. doi: 10.1038/s41467-026-73214-w (PMC13194983; doi:10.1038/s41467-026-73214-w)
Supplement: Supplementary file 1 — Supplementary information [file 41467_2026_73214_MOESM1_ESM.pdf]

Supporting information for:

Over four minutes of pyruvate T_1 using chemically and physically induced deceleration of relaxation

Josh P. Peters[a], Florin Teleanu[b,c], Huijing Zou[b], Ehtisham Rasool[a], Farhad Haj Mohamad[a], Heiner Schäfer[d], Jan-Bernd Hövener[a], Alexej Jerschow[b], Andrey N. Pravdivtsev[a]†

[a] Section Biomedical Imaging (SBMI), Molecular Imaging North Competence Center (MOINCC), Department of Radiology and Neuroradiology, University Hospital Schleswig-Holstein, Kiel University, Am Botanischen Garten 14/18, 24118 Kiel, Germany. E-mail: andrey.pravdivtsev@rad.uni-kiel.de

[b] Department of Chemistry, New York University, New York, NY 10003, United States. E-mail: aj39@nyu.edu

[c] ELI-NP, “Horia Hulubei” National Institute for Physics and Nuclear Engineering, 30 Reactorului Street, Bucharest-Magurele, 077125, Ilfov, Romania

[d] Institute for Experimental Cancer Research, Medical Faculty, Kiel University, Haus K3, Arnold-Heller-Str. 3, 24105 Kiel

†Corresponding authors: josh.peters@rad.uni-kiel.de, andrey.pravdivtsev@rad.uni-kiel.de

Contents

Supplementary Note 1. Material for Methods 2
Supplementary Note 2. Extracted T_1-NMRD 4
Supplementary Note 3. Hierarchy of MD simulation 6
Supplementary Note 4. ^{13}C pyruvate longitudinal relaxation in two-site chemical exchange ...10
Supplementary Note 5. Data analysis and fitting models 12
Supplementary Note 6. Pyruvate to pyruvate hydrate ratio as a function of temperature........15
Supplementary Note 7. Chemical exchange rate between pyruvate and pyruvate hydrate.....17
Supplementary Note 8. References (Supporting Information) 18

Supplementary Note 1. Additional Material for Methods

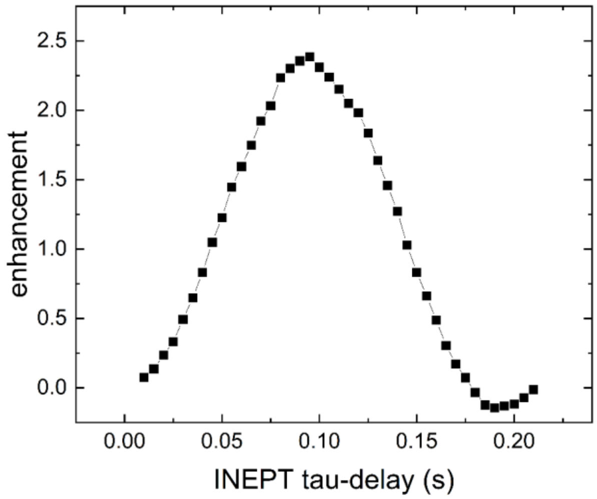

Fig. S1: INEPT calibration curve to find the optimum value for the interpulse delay (2×tau) to transfer the ^{1}H polarization to the 1-^{13}C of pyruvate. *A maximum enhancement compared to the thermal ^{13}C spectrum of 2.4 is visible at a tau-delay of 96 ms. Such a calibration was performed for each sample except for the one with deuterated pyruvate. This value corresponds well to the theoretically expected: τ=1/8 J, with J~1.3 Hz. Measured data (black squares) are connected to guide the eye (black line). The curve has been recorded once for each sample (n=1 independent experiments). Source data are provided as a Source Data file.*

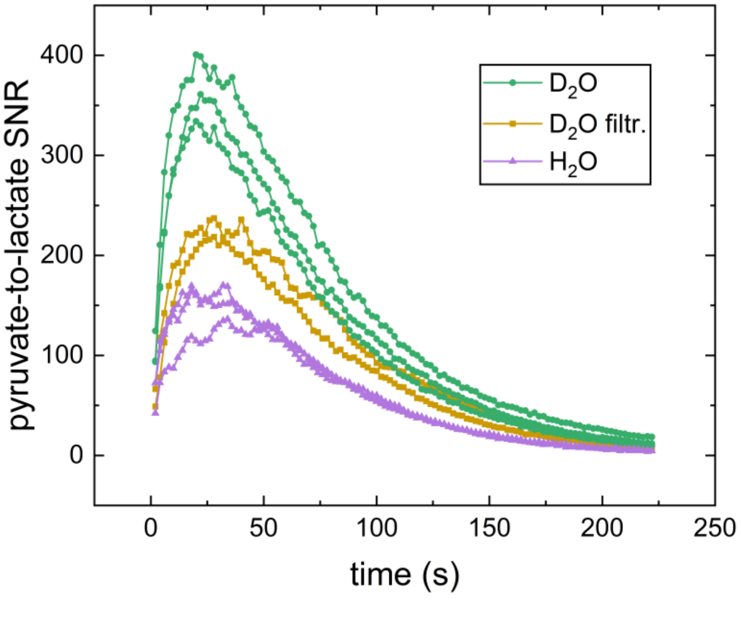

Fig. S2: SNR during the metabolic experiments performed to obtain Figure 4 in the main text. *Three dissolution media were used, leading to highest SNR in the case of optimized sample #C without filtration step (D_2O, green circles), medium SNR with filtration (D_2O filtr., orange squares), and lowest SNR without optimization (H_2O, lila triangles). Datappints have been*

connected to guide the eye. Each curve has been recorded once (n=1 independent experiments). Source data are provided as a Source Data file.

Tab. S1: *Sample compositions for samples A-I and corresponding T_1 values at low (7.8 µT to 8 mT), medium (96 to 1024 mT), and high fields (9.4 T). The table is also supplied as an Excel sheet.*

#	pH	H2O (%)	D2O (%)	size (µL)	heated in DM?	degassed?	[pyruvate] (mM)	pyruvate	[trityl] (µM)	[Tris buffer] (mM)	[EDTA] (mM)	[NaCl] (mM)	[Vitamin C] (mM)	low field (s)	stdev (s)	medium field (s)	stdev (s)	high field (s)	high stdev (s)
A	7.69	0	100	300	no	yes	90.14	1-13C, d4	0	57.15	4.76			224.52	8.80	231.09	10.89	83.93	1.22
B	7.60	0	100	550	no	yes	90.04	1-13C	0	45.19	3.88			187.97	4.06	193.11	4.58	80.70	1.01
C	7.86	0	100	550	yes, filtered	yes	98.90	1-13C	200	40	0.27	50	2.46	172.20	3.11	178.13	1.19	76.42	0.70
D	7.60	0	100	550	no	no	90.04	1-13C	0	45.19	3.88			84.23	0.86	93.34	7.50	64.37	0.44
E	7.58	90	10	550	no	no	89.25	1-13C	0	44.63	4.38			51.50	0.59	54.66	2.54	46.85	0.28
F	7.68	90	10	550	no	no	89.81	1-13C	0	42.51				49.96	0.92	52.30	2.11	45.65	0.39
G	7.33	90	10	550	yes	no	98.60	1-13C	173	40	0.27	50		46.55	1.36	55.40	2.19	45.68	0.43
H	7.55	0	100	550	no	no	90.75	1-13C	0					43.39	1.50	73.73	6.54	55.95	0.88
I	7.60	90	10	550	no	no	90.94	1-13C	0					30.90	0.93	48.64	2.47	41.84	0.37

Supplementary Note 2. Extracted T_1-NMRD

Interestingly, we found a second "relaxation" component during NMRD measurements of pyruvate (see examples in Fig. S3). This second relaxation component was always much faster than the reported ^{13}C relaxation time here: it ranged from 1.1 to 8.2 (Tab. S2). The longest second relaxation component was found for sample #B, which also has a long T_1. For sample #A**,** no two-exponential behavior was observed, hence it was fit with a regular one-exponential curve. We had two hypotheses on the origin of the effect: diffusion of the sample during field cycling and polarization transfer from protons to ^{13}C. However, the measurements do not confirm this effect, as the amplitude was shown to be deuteration-independent (hence no polarization transfer from protons) and did not show a perfect correlation with the relaxation time (hence unlikely to be diffusion). The amplitude of this component was typically less than 2% of the main component's amplitude and could therefore be ignored by skipping some of the first points of kinetics. However, for simplicity, we opted for the two-exponential fitting. When checking for the difference between two- and one-exponential fitting, an average absolute deviation in T_1 of only (2.36±2.14)% was observed across all samples and fields.

Tab. S2: T_1 nuclear magnetic resonance dispersion curves for the samples #A-I*. Note that a bi-exponential curve fit was used, and the second relaxation component was shared across all fields, while the other parameters were free. For sample #A**,** no second component could be observed; hence, it was fitted with a single-exponential curve. The table is also supplied as an Excel sheet.*

#	A		B		C		D		E		F		G		H		I	
2nd relax. comp. value / stdev (s)	mono	mono	8.16	1.77	2.68	0.68	1.07	0.12	1.24	0.12	3.40	0.46	2.84	0.76	3.85	0.35	3.87	0.26
magnetic field (T)	T_1 (s)	T_1 stdev (s)	T_1 (s)	T_1 stdev (s)	T_1 (s)	T_1 stdev (s)	T_1 (s)	T_1 stdev (s)	T_1 (s)	T_1 stdev (s)	T_1 (s)	T_1 stdev (s)	T_1 (s)	T_1 stdev (s)	T_1 (s)	T_1 stdev (s)	T_1 (s)	T_1 stdev (s)
7.81E-06	208.21	8.65	186.63	6.10	167.63	2.86	85.70	0.97	51.02	0.47	48.01	0.61	46.20	0.57	45.29	1.19	31.05	0.40
1.56E-05			190.62	6.24			83.06	0.91	50.53	0.47	48.71	0.63			41.68	1.00	30.48	0.39
3.13E-05	220.90	9.10	192.38	6.37	174.97	2.87	85.24	0.94	51.43	0.47	48.90	0.63	44.60	0.54	43.09	1.04	29.32	0.37
6.25E-05			184.71	5.90			83.18	0.92	51.19	0.47	49.24	0.63			41.88	1.01	30.61	0.39
9.38E-05			196.49	6.55			83.84	0.92	50.52	0.46	49.57	0.64			42.73	1.03	30.09	0.38
1.25E-04	226.41	9.46	183.44	5.81	168.40	2.70	84.03	0.93	50.76	0.46	49.40	0.64	45.17	0.55	42.31	1.01	30.62	0.39
1.88E-04			184.60	5.95			84.72	0.94	52.05	0.48	50.02	0.65			43.52	1.06	30.71	0.39
2.50E-04			186.73	5.98			85.73	0.96	50.98	0.47	50.45	0.66			43.76	1.07	30.41	0.39
3.75E-04			193.94	6.43			84.03	0.93	51.52	0.48	51.97	0.72			43.39	1.04	30.97	0.40
5.00E-04	237.36	10.17	184.64	5.95	175.81	2.94	82.64	0.91	52.05	0.48	49.89	0.65	47.45	0.59	42.71	1.02	30.43	0.39
7.50E-04			182.17	5.86			84.37	0.94	52.13	0.49	50.39	0.66			42.15	1.01	30.61	0.39
0.001			185.83	6.08			84.58	0.95	51.90	0.49	50.05	0.64			42.37	1.03	30.29	0.39
0.0015			182.99	5.96			83.27	0.93	51.95	0.49	49.87	0.64			42.25	1.02	30.64	0.40
0.002	228.45	9.66	187.70	6.17	173.40	2.85	84.09	0.94	51.63	0.49	49.68	0.64	47.37	0.58	43.10	1.04	30.79	0.40
0.003			189.80	6.18			83.71	0.93	51.42	0.48	50.16	0.66			43.69	1.06	31.42	0.40
0.004			194.10	6.52			85.05	0.95	52.78	0.50	50.96	0.67			43.44	1.04	31.32	0.40
0.006			188.90	6.18			84.80	0.94	51.74	0.48	51.04	0.70			45.93	1.11	33.30	0.43
0.008	225.82	9.80	187.84	6.17	172.98	2.86	84.20	0.93	51.37	0.48	50.89	0.66	48.52	0.61	47.74	1.15	33.09	0.43
0.012			197.13	6.69			83.38	0.93	51.35	0.47	49.40	0.63			50.06	1.17	37.91	0.49
0.016			181.47	5.72			84.52	0.94	51.67	0.47	50.30	0.64			52.34	1.25	38.16	0.48
0.024			192.98	6.44			84.49	0.93	52.45	0.49	49.83	0.63			57.67	1.41	41.20	0.53
0.032	221.63	9.25	189.66	6.32	176.18	2.94	84.17	0.95	52.30	0.48	49.89	0.63	49.48	0.59	59.16	1.43	41.61	0.53
0.048			192.30	6.36			85.04	0.94	51.57	0.47	50.18	0.64			62.16	1.54	44.13	0.58
0.064	232.08	9.77	196.26	6.64	174.60	2.92	85.58	0.95	52.30	0.48	50.55	0.64	52.35	0.63	64.94	1.64	44.15	0.58
0.096			195.11	6.66			86.28	0.95	52.61	0.49	49.61	0.62			67.55	1.68	46.38	0.61
0.128	240.14	10.02	193.26	6.49	177.58	2.96	85.91	0.96	52.01	0.48	49.71	0.63	52.64	0.63	66.32	1.65	45.03	0.58
0.192			197.35	6.70			89.23	1.00	52.55	0.48	51.99	0.67			68.57	1.72	47.34	0.63
0.256	225.49	8.99	185.45	5.97	178.00	2.99	89.06	1.02	53.10	0.49	51.23	0.65	53.05	0.63	69.61	1.76	47.24	0.62
0.384			189.83	6.18			91.28	1.03	54.29	0.49	51.78	0.65			73.33	1.85	49.04	0.66
0.512	242.10	9.41	188.20	6.06	180.01	2.99	94.34	1.08	55.61	0.51	53.57	0.68	56.06	0.66	77.96	2.01	49.65	0.65
0.768	235.12	9.27	199.76	6.67	176.41	2.87	102.34	1.24	57.50	0.52	54.59	0.69	57.08	0.66	81.60	2.08	52.66	0.70
1.024	212.59	7.61	195.97	6.32	178.66	2.91	108.28	1.34	59.60	0.54	55.95	0.70	58.16	0.66	84.93	2.19	51.75	0.66
1.536	226.48	7.96	189.17	5.83	167.84	2.54	114.55	1.44	61.36	0.55	58.22	0.71	60.50	0.68	91.51	2.32	54.95	0.71
2.048	226.34	7.60	180.60	5.17	166.48	2.46	117.46	1.48	62.52	0.54	59.28	0.72	60.06	0.66	90.44	2.24	53.27	0.65
3.072	212.27	6.36	169.72	4.33	156.40	2.13	114.88	1.34	61.81	0.52	58.75	0.68	59.44	0.62	89.08	2.08	54.24	0.65
4.096	173.93	5.65	154.98	3.45	143.04	1.80	106.20	1.13	61.20	0.48	57.80	0.64	58.12	0.58	83.73	1.83	52.71	0.60
6.144	132.88	2.63	123.32	2.07	114.43	1.23	91.02	0.80	55.93	0.40	54.00	0.55	53.19	0.50	75.44	1.48	49.31	0.51
8.192	102.54	1.70	95.43	1.35	89.41	0.85	74.39	0.56	50.69	0.33	48.34	0.44	48.95	0.43	63.51	1.10	44.43	0.41
9.4	83.93	1.22	80.70	1.01	76.42	0.70	64.37	0.44	46.85	0.28	45.65	0.39	45.68	0.43	55.95	0.88	41.84	0.37

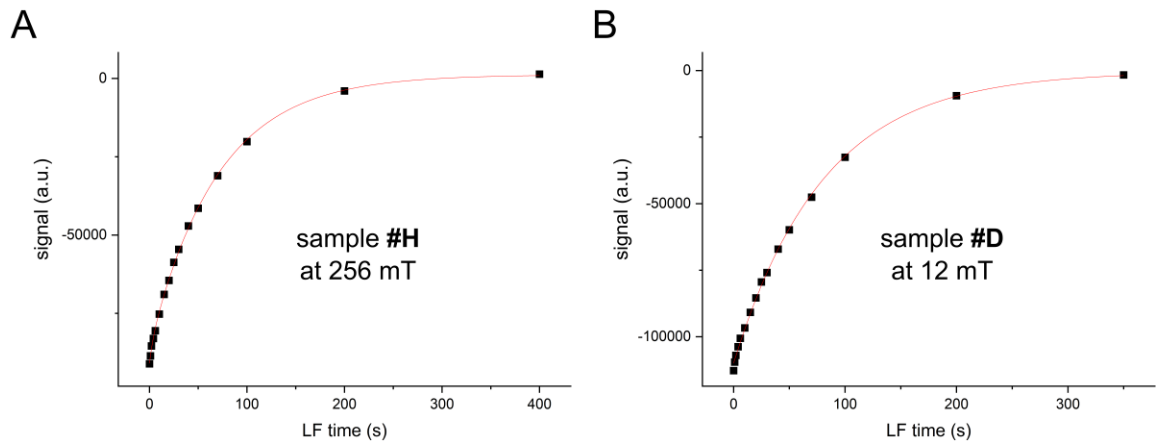

Fig. S3: Slight two-exponential behavior of samples #H and #D from Fig. 2 in the main text. *A slight two-exponential behavior can be observed for the first few points. The amplitude of the second component is typically less than 2% of the first exponential component. Measured datapoints (black squares) and two-exponential fit (red line). Each curve has been recorded once (n=1 independent experiments). Source data are provided as a Source Data file.*

Supplementary Note 3. Hierarchy of MD simulation

The MD simulation pipeline is illustrated in Fig. S4 and described in detail below.

System preparation: The initial molecular geometries of the Py and PyH molecules were optimized using Gaussian16 Frisch et al.[1] at the B3LYP/def2TZVPP level of theory.

The initial box for MD contained one pyruvate molecule, Py, one pyruvate hydrate molecule, PyH, and 1274 water molecules. Two Na^+ ions were added to neutralize the overall charge of the system. The Packmol package randomly distributed these molecules inside a 34 Å cubic box. A minimum intermolecular distance (tolerance) of 2.0 Å was imposed to reduce atomic overlaps during packing. The generated coordinates were written in PDB format. For averaging purposes, we generated 10 statistically independent initial configurations while preserving the same system composition and box size. Subsequent MD simulations have been performed with GROMACS package Abraham et al.[2]. The optimized structures were processed through CGenF Vanommeslaeghe et al.[3] to obtain CHARMM36-compatible force-field parameters used for the Py and PyH molecules, while the TIP4P force field was used for water molecules.

Energy minimization: A preliminary steepest-descent energy relaxation was performed to relieve steric crowding and dissipate unfavorable contacts embedded in the starting coordinates. An initial energy relaxation was performed via steepest-descent minimization (≤ 500 steps or until $F_{max} < 1000$ kJ mol^{-1} nm^{-1}) to eliminate unfavorable contacts and yield a low-energy, clash-free conformation suitable for subsequent dynamics.

System Equilibration:

NVT Equilibration: The energy-minimized simulation box was equilibrated under NVT conditions using GROMACS with a 2 fs time step for 50,000 steps (100 ps). Independent runs were performed with the thermostat set to temperatures ranging from 293 to 353 K in 10 K increments. A set of 10 independent runs was performed at each temperature using the velocity-rescale thermostat with a coupling constant of 0.1 ps. All bonds involving hydrogen atoms were constrained using LINCS. Short-range van der Waals and electrostatic interactions were truncated at 1.2 nm, with long-range electrostatics treated using the PME method. Periodic boundary conditions were applied in all directions, and no pressure coupling was used.

NPT Equilibration: The resulted system was equilibrated for 100 ps under NPT conditions with position restraints applied. Simulations used a 2 fs leap-frog timestep with LINCS constraints on all hydrogen bonds. The thermostat was set to temperatures ranging from 293 to 353 K in 10 K increments. A set of 10 independent runs was performed at each temperature (70 runs in total). Each NPT equilibration used a velocity-rescale thermostat and C-rescale barostat at 1 bar. Electrostatics were treated with PME, while Lennard-Jones interactions used a 1.0 to 1.2 nm force-switch cutoff.

MD Production: The production simulation was performed under NPT conditions using a standard leap-frog integrator, taking 1 million steps at 1 fs with the gentle V-rescale thermostat, allowing the box to expand naturally at 1 bar with a matching barostat. The thermostat was set to temperatures ranging from 293 to 353 K in increments of 10 K. A set of 10 independent runs was

performed at each temperature. The total length of the production run was 1 ns. Coordinates were saved every 1 ps to balance resolution with data efficiency.

Data Interpretation: From the MD production trajectories, we've computed the autocorrelation functions of the time-dependent intra- and inter-molecular ^{13}C-^{1}H dipolar interactions in order to estimate their corresponding spectral densities. Then, the intra- and intermolecular contributions to the longitudinal relaxation rate of the carboxylate carbon atom of both Py and PyH, and their corresponding correlation times, were predicted. The predicted values were computed with a custom script and averaged across the 10 production runs. Results are summarized in Tab. S3. For the case of intramolecular ^{1}H-^{13}C dipole-dipole relaxation, the relaxation rate contribution is given by

$$R_1^{intraDD} = \frac{1}{4}\left(\frac{\mu}{4\pi}\hbar\gamma_H\gamma_C\frac{1}{r_{CH}^3}\right)^2 \left(J(\omega_H - \omega_C) + 3J(\omega_C) + 6J(\omega_H + \omega_C)\right)$$

where the μ is representing the permeability of the vacuum, $\hbar$ is the reduced Planck constant, the γ_H and γ_C are the gyromagnetic ratios of the hydrogen and carbon, the ω_H and the ω_C are the corresponding Larmor frequencies and r_{CH} is the intramolecular proton-carbon distance. The spectral density $J(\omega)$ characterizing the intramolecular ^{1}H-^{13}C dipole-dipole interaction is defined as the real part of the Fourier transform of the corresponding autocorrelation function $G^{intraDD}(t)$ computed from the MD trajectory. The correlation time $\tau_C^{intraDD}$ is extracted by fitting the autocorrelation function using a single exponential $G^{intraDD}(t) = A \cdot \exp\left(-\frac{t}{\tau_C^{intraDD}}\right)$. A similar approach is used for the intermolecular dipolar contribution.

For estimating the high-field CSA contribution, we've assumed that the correlation time of the CSA interaction is the same as for the intra-molecular dipolar interactions, as both are modulated by rotational diffusion ($\tau_C^{CSA} \sim \tau_C^{intraDD} \sim \tau_C^{rot}$). The chemical shift anisotropy, $\Delta\sigma$, was estimated from DFT simulations of the chemical shielding tensor using ORCA at the B3LYP def2-TZVP level of theory for a single Py molecule in the gas phase whose structure was previously optimized at the same level of theory. The CSA contribution to the longitudinal relaxation rate is given by $R_1^{CSA} = \frac{(\Delta\sigma\gamma_C B_0)^2}{3} J(\omega_C)$ where B_0 is the main external field and the spectral density is assumed to be a simple Lorenzian function $J(\omega) = \frac{2}{5}\frac{\tau_C^{rot}}{1+(\omega\tau_C^{rot})^2}$.

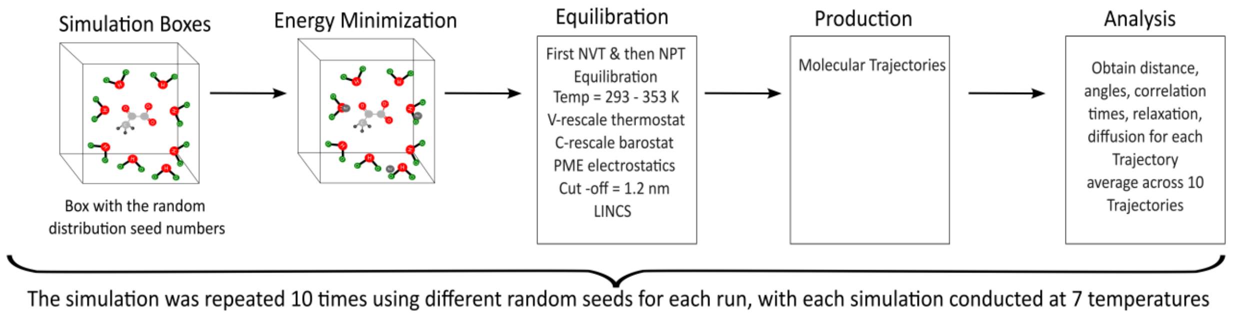

Fig. S4. MD simulation pipeline. *Simulation of Py and PyH in a water box. (step 1, simulation boxes) 10 simulation boxes were generated by using different seeding numbers. (step 2, energy minimization) Energy minimization was applied and completed once the maximum internal force converged below 1000 kJ mol^{-1} nm^{-1}. (step 3, equilibration) The system was equilibrated at specific temperatures using NVT and NPT thermostats. (step 4, production) Molecular trajectories of 1 ns length were calibrated for 10 boxes and 7 temperatues yielding 70 trajectories. (step 5, analysis) Extraction of autocorrelation functions of the intra- and inter-molecular ^{1}H-^{13}C dipolar interactions, correlation times, and calculation of relaxation contributions. Follow-up DFT calculations were used to estimate the CSA factor $\Delta\sigma$ for a single Py molecule in gas phase.*

***Tab. S3**. Longitudinal relaxation rates of carboxylate ^{13}C due to either intra- or intermolecular ^{1}H-^{13}C dipolar couplings for Py and PyH species as predicted from MD simulations (dataset used for the plot of Fig. 3B in the main text).*

Temperature (K)	$R_1^{\mathrm{Py,interDD}(^1\mathrm{H})}$ (s^{-1})		$R_1^{\mathrm{Py,intraDD}(^1\mathrm{H})}$ (s^{-1})		$R_1^{\mathrm{PyH,interDD}(^1\mathrm{H})}$ (s^{-1})		$R_1^{\mathrm{PyH,intraDD}(^1\mathrm{H})}$ (s^{-1})	
	average	STD	average	STD	average	STD	average	STD
293	0.00580	0.00050	0.00068	0.00017	0.00576	0.00045	0.00481	0.00099
303	0.00467	0.00030	0.00055	0.00012	0.00483	0.00036	0.00359	0.00070
313	0.00393	0.00027	0.00044	0.00012	0.00404	0.00012	0.00272	0.00033
323	0.00326	0.00009	0.00039	0.00009	0.00338	0.00017	0.00232	0.00023
333	0.00283	0.00015	0.00034	0.00012	0.00293	0.00010	0.00209	0.00032
343	0.00238	0.00009	0.00028	0.00006	0.00246	0.00011	0.00178	0.00027
353	0.00211	0.00012	0.00028	0.00005	0.00218	0.00006	0.00151	0.00014

***Tab. S4**. Correlation times of either intra- or intermolecular ^{1}H-^{13}C dipolar interactions for Py and PyH species as predicted from MD simulations*

Temperature (K)	$\tau_\mathrm{C}^{\mathrm{Py,interDD}}$ (ps)		$\tau_\mathrm{C}^{\mathrm{Py,intraDD}}$ (ps)		$\tau_\mathrm{C}^{\mathrm{PyH,interDD}}$ (ps)		$\tau_\mathrm{C}^{\mathrm{PyH,intraDD}}$ (ps)	
	average	STD	average	STD	average	STD	average	STD
293	7.476	0.651	7.310	2.220	7.021	0.593	10.813	2.780
303	6.133	0.402	5.594	1.305	6.516	0.464	9.293	1.451
313	5.080	0.407	4.743	1.760	4.939	0.197	5.773	0.791
323	4.228	0.188	4.027	0.984	4.142	0.212	5.025	0.621
333	3.754	0.244	3.398	1.665	3.643	0.126	4.488	0.675
343	3.085	0.103	2.545	0.656	3.053	0.190	3.835	0.693
353	2.775	0.173	2.594	0.540	2.661	0.063	3.235	0.337

Supplementary Note 4. ^{13}C pyruvate longitudinal relaxation in two-site chemical exchange

The ^{13}C and ^{1}H spectra of the pyruvate solution show two peaks corresponding to the oxo (Py) and hydrated (PyH) forms, which undergo chemical exchange ($\mathrm{Py} \leftrightarrow \mathrm{PyH}$) with a corresponding equilibrium constant $K_{\mathrm{eq}} = \frac{p_{\mathrm{PyH}}}{p_{\mathrm{Py}}}$. We assume that the conversion is slow compared to the timescale of signal acquisition but fast compared to the polarization decay rates. By measuring the ratio of the two peak integrals as a function of temperature, we observe that at room temperature the equilibrium constant of the exchange process is $K_{eq} = 0.1$ and decreases with increasing temperature (Fig. S5A).

The PyH form includes two extra protons from the hydroxyl groups, leading to additional intramolecular ^{1}H-^{13}C dipolar couplings. Based on our MD predictions (Tab. S3 and Fig. 5B in the Main Text), the intramolecular dipolar rates for PyH are of the same order as the intermolecular contributions and around 7 times larger than the intramolecular rates in Py. Thus, we expect that the two pyruvate species will have sizeably different ^{13}C lifetimes. The CSA and PRE contributions are assumed to be identical for the two species.

The system of coupled differential equations describing both longitudinal relaxation and chemical exchange is

$$\frac{d}{dt}\begin{pmatrix} M_{\mathrm{z}}^{\mathrm{Py}}(t) \\ M_{\mathrm{z}}^{\mathrm{PyH}}(t) \end{pmatrix} = \begin{pmatrix} -R_1^{\mathrm{Py}} - k_1 & k_{-1} \\ k_1 & -R_1^{\mathrm{PyH}} - k_{-1} \end{pmatrix} \begin{pmatrix} M_{\mathrm{z}}^{\mathrm{Py}}(t) - M_{\mathrm{z,eq}}^{\mathrm{Py}} \\ M_{\mathrm{z}}^{\mathrm{PyH}}(t) - M_{\mathrm{z,eq}}^{\mathrm{PyH}} \end{pmatrix} \quad \textbf{Eq. S1}$$

where $R_1^{\mathrm{Py\ or\ PyH}}$ are the site-specific longitudinal relaxation rates, k_1 and k_{-1} are the forward ($\mathrm{Py} \overset{k_1}{\rightarrow} \mathrm{PyH}$) and backward ($\mathrm{PyH} \overset{k_{-1}}{\longrightarrow} \mathrm{Py}$) reaction rates connected as $K_{\mathrm{eq}} = k_1/k_{-1} = p_{\mathrm{PyH}}/p_{\mathrm{Py}}$ and $M_{\mathrm{z,eq}}^{\mathrm{Py\ or\ PyH}}$ are the equilibrium longitudinal polarization of the two species connected as $M_{\mathrm{z,eq}}^{\mathrm{Py}}/M_{\mathrm{z,eq}}^{\mathrm{PyH}} = p_{\mathrm{Py}}/p_{\mathrm{PyH}}$. The system of equations is solved analytically (assuming $K_{eq} = 0.1$) in the attached Mathematica notebook for the case of non-selective inversion recovery experiments ($M_{\mathrm{z}}^{\mathrm{Py}}(0) = -M_{\mathrm{z,eq}}^{\mathrm{Py}}$; $M_{\mathrm{z}}^{\mathrm{PyH}}(0) = -M_{\mathrm{z,eq}}^{\mathrm{PyH}}$).

In Fig. S5B, we plot the recovery profiles corresponding to either the Py or PyH auto-relaxation rates, as well as the inversion profile described by the population-weighted average rate (no chemical exchange). The solutions of Eq. S1 for $M_{\mathrm{z}}^{\mathrm{Py}}(t)$ undergo both recovery and chemical exchange, and it lies in the shaded region. For exchange rates much slower than the decay rates, $M_{\mathrm{z}}^{\mathrm{Py}}(t)$ follows a recovery process described by the R_1^{Py} rate. For the case when $\left(|k_1 - k_{-1}| \gg \left|R_1^{\mathrm{Py}} - R_1^{\mathrm{PyH}}\right|\right)$, the observed recovery profile monitored at the Py peak follows a standard inversion recovery profile with an apparent rate equal to the population-weighted average of the rates corresponding to the two species: $R_1^{\mathrm{avg}} = p_{\mathrm{Py}} R_1^{\mathrm{Py}} + p_{\mathrm{PyH}} R_1^{\mathrm{PyH}}$ with $p_{\mathrm{Py}}\!:\!p_{\mathrm{PyH}} = 10\!:\!1$ at room temperature. We use this result to compare MD-derived dipolar relaxation rates with experimental values.

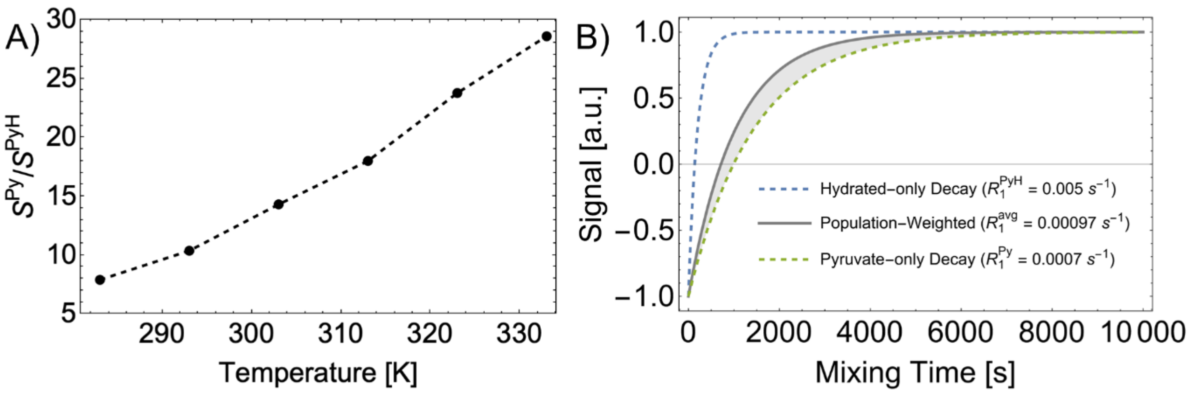

Fig. S5. *A) Experimental signal integral ratio of the two Py (@170.3 ppm) and PyH (178.7 ppm) peaks in ^{13}C spectrum at different temperatures highlighting the shift of the chemical equilibrium for two species in water. B) Inversion recovery profile described by the corresponding relaxation rates. The solution of Eq. S1 describing* $M_z^{Py}(t)$ *undergoing both recovery and chemical exchange lies in the shaded gray region for exchange rate constants* $k_{ex} = \frac{k_1}{p_{Py}} = \frac{k_{-1}}{p_{PyH}}$ *between* $10^{-5} - 10^{-1}\ s^{-1}$. *Source data are provided as a Source Data file.*

Supplementary Note 5. Data analysis and fitting models

To isolate and quantify individual relaxation contributions, we have subtracted pairs of datasets that differ in only one chemical/physical feature. The following steps were done to unveil relaxation contributions.

- The background contribution (R_1^0) was estimated as the averaged low-field relaxation rate of sample #A. This comes from intra- and inter-molecular ^{2}H-^{13}C dipolar couplings as well as other mechanisms that couldn't be separated, like spin rotation. The contribution of the former is computed from MD simulations as

$$R_1^{0,{}^2\mathrm{H}-{}^{13}\mathrm{C}} = p_{\mathrm{Py}}\left(R_1^{\mathrm{Py,intraDD}({}^2\mathrm{H})} + \mathrm{R}_1^{\mathrm{Py,interDD}({}^2\mathrm{H})}\right) + p_{\mathrm{PyH}}\left(R_1^{\mathrm{PyH,intraDD}({}^2\mathrm{H})} + \mathrm{R}_1^{\mathrm{PyH,interDD}({}^2\mathrm{H})}\right)$$

  where we calculate the deuterium relaxation contributions from Tab. S3 by using the scaling factor $R_1^{2\mathrm{H}}/R_1^{1\mathrm{H}} = S_{2\mathrm{H}}(S_{2\mathrm{H}}+1)\gamma_{2\mathrm{H}}^2/S_{1\mathrm{H}}(S_{1\mathrm{H}}+1)\gamma_{1H}^2$ where S_H and γ_H are the nuclear spin and the gyromagnetic ratio values of the corresponding hydrogen isotope.

- The chemical shield anisotropy contribution rate, R_1^{CSA}, was extracted by fitting the high-field region of sample #A data, assuming a fast-tumbling regime ($\omega_{13C}\tau_{\mathrm{C}}^{\mathrm{CSA}} \ll 1$), such that the dispersion profile is given by the quadratic dependence of the CSA interaction with respect to the magnetic field.

- The intramolecular ^{1}H-^{13}C dipolar contribution, R_1^{intraDD}, was estimated by subtracting the two datasets corresponding to samples #A ([1-^{13}C]pyruvate-d$_4$) and #B ([1-^{13}C]pyruvate). The correlation time for this interaction, evaluated from MD simulations, was smaller than 10 ps for all temperatures, further supporting our evaluation that spin interactions modulated by molecular rotation (intraDD and CSA) are in a fast-tumbling regime ($\omega_{13C}\tau_{\mathrm{C}}^{\mathrm{rot}} \ll 1$), on the whole range of measurement fields (10^{-5} – 9.4 T).

- The intermolecular ^{1}H-^{13}C dipolar contribution (interDD) was estimated by subtracting either the two datasets corresponding to samples #D ([1-^{13}C]pyruvate in D_2O + chelating agents) and #E ([1-^{13}C]pyruvate in H_2O + chelating agents), or the two datasets corresponding to samples #H ([1-^{13}C]pyruvate in D_2O) and #I ([1-^{13}C]pyruvate in H_2O). From MD simulations, we evaluate the translational correlation time of this interaction and obtained values smaller than 10 ps for all temperatures ($\omega_{13C}\tau_{\mathrm{C}}^{\mathrm{trans}} \ll 1$), suggesting that the interDD contribution is constant and field-independent.

- The paramagnetic relaxation enhancement due to molecular oxygen (PRE O_2) was estimated by subtracting either the two datasets corresponding to samples #D ([1-^{13}C]pyruvate) and #B (degassed [1-^{13}C]pyruvate). Fig. S6A clearly shows a field-dependency which was fitted with the following equation Kowalewski et al.[4]:

$$R_1^{\mathrm{PRE}} = \frac{32\pi}{405}\left(\frac{\mu}{4\pi}\hbar\gamma_{\mathrm{e}}\gamma_{\mathrm{C}}\right)^2 \frac{S(S+1)}{d\cdot D_{12}} 1000 N_{\mathrm{A}} C_{\mathrm{M}}\left(7J_{\mathrm{inter}}(\omega_e) + 3J_{\mathrm{inter}}(\omega_C)\right) \qquad \textbf{Eq. S2}$$

  where μ is the magnetic permittivity of the vacuum, $\hbar$ is the reduced Planck constant, γ_{e} and γ_{C} are the gyromagnetic ratios of the electron and carbon spin, respectively, *S* is the electronic spin of the molecular oxygen (triplet), *d* is the distance of closest approach between oxygen and pyruvate's C1 atom, D_{12} is the sum of the self-diffusion rates of pyruvate and molecular oxygen, N_{A} is Avogadro's number, C_{M} is the molar concentration

of dissolved oxygen in normal conditions and $J_{\text{inter}}(\omega)$ is the intermolecular spectral density Hwang et al.[5] given by:

$$J_{\text{inter}}(z) = \frac{1+\frac{5z}{8}+\frac{z^2}{8}}{1+z+\frac{z^2}{2}+\frac{z^3}{6}+\frac{4z^4}{81}+\frac{z^5}{81}+\frac{z^6}{648}}, \text{ with } z = \left(2\omega\tau_{\text{C}}^{\text{PRE}}\right)^{0.5} \qquad \textbf{Eq. S3}$$

Our fitted model (red line in Fig. S6A) assumes a constant fitting prefactor and matches well the experimental field dispersion. The fitted correlation time of the PRE interaction is $\tau_{\text{C}}^{\text{PRE O}_2}$ = 6.12 ± 1.95 ps, in good agreement with previous reports Teng et al.[6].

- Additionally, we attempted to estimate oxygen concentration by fitting R_1^{PRE} (Eq. S2) to experimental data using diffusion rates and electron relaxation time of molecular oxygen, and the distance of closest approach was estimated as equal to the sum of Van der Waals radii of O and C: $d = r_{\text{O}_2}^{\text{VdW}} + r_{\text{Py}}^{\text{VdW}} \sim r_{\text{O}}^{\text{VdW}} + r_{\text{C}}^{\text{VdW}}$ (Tab. S5). This yielded a concentration of $C_{\text{M}}^{\text{O}_2} \sim 140\ \mu\text{M}$, in reasonable agreement with other results Livo et al.[7].

Tab. S5. Values used to predict the $R_1^{\text{PRE O}_2}$ using **Eq. S2**.

$r_{\text{O}_2}^{\text{VdW}}$ [A]	$r_{\text{Py}}^{\text{VdW}}$ [A]	D_{O_2} [m^2s^{-1}]	D_{Py} [m^2s^{-1}]	$T_{1e}^{O_2}$ [ps]
1.5	1.7[a]	2×10^{-9}[b]	1.15×10^{-9}[c]	6.8[d]

[a] [Bondi[8],; [b] Jamnongwong et al.[9], Koelsch et al.[10], Teng et al.[6]

- The additional paramagnetic relaxation enhancement was assigned to a paramagnetic transition metal cation (PRE M^+) given the additional shoulder in the NMRD profile of samples #I and #H, which disappears upon chelation with Tris, EDTA, or both (samples #E, #F, and #G). This indicates an extra PRE characterized by a larger correlation time of several hundred picoseconds. The field-dispersion of the PRE M^+ contribution is clear by subtracting either samples #H ([1-^{13}C]pyruvate in D_2O) and #D ([1-^{13}C]pyruvate in D_2O + chelating agents), or samples #I ([1-^{13}C]pyruvate in H_2O) and #E ([1-^{13}C]pyruvate in H_2O + chelating agents). The nature of the metal cation cannot be inferred from current data, so we fitted the PRE M^+ dispersion profiles assuming a constant prefactor in Eq. S2 (red lines in Fig. S6B and C). The analytical equation fits both datasets decently, with an extracted correlation time of $\tau_{\text{C}}^{\text{PRE M}^+}$ = 414 ± 69 ps and $\tau_{\text{C}}^{\text{PRE M}^+}$=557 ± 114 ps, respectively.
- The Mathematica notebook summarizing all these calculations is attached as Supporting Information.

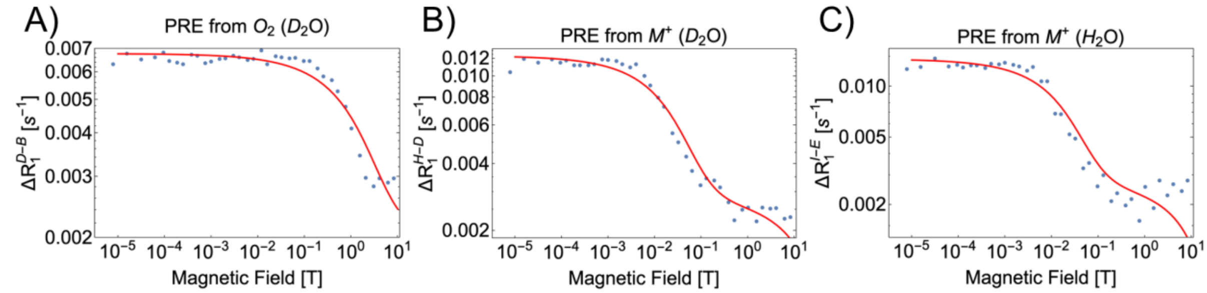

***Fig. S6**. Field-dependent nuclear magnetic relaxation disperison profiles of paramagnetic relaxation enhancement (PRE) contributions for molecular oxygen (A) and transition metal cation (B & C) derived by subtracting selected pairs of experimental datasets from Fig. 2A of Main Text. Data points were fitted using Eq. S2 assuming a constant prefactor* $A_0 = \frac{32\pi}{405}\left(\frac{\mu}{4\pi}\hbar\gamma_e\gamma_C\right)^2 \frac{S(S+1)}{d\cdot D_{12}} 1000 N_A C_M$ *leading to the following fitted values:* $\tau_C^{PRE\ O_2}$ = 6.12 ± 1.95 ps, $A_0^{PRE\ O_2} = 6.57\cdot 10^{-4} \pm 0.58\cdot 10^{-4}\ s^{-1}$ (A); $\tau_C^{\mathrm{PRE\ M^+}}$ = 414 ± 69 ps, $A_0^{PRE\ \mathrm{M^+}} = 1.34\cdot 10^{-3} \pm 0.37\cdot 10^{-3}\ s^{-1}$ (B); $\tau_C^{\mathrm{PRE\ M^+}}$= 557 ± 114 ps, $A_0^{PRE\ \mathrm{M^+}} = 1.68\cdot 10^{-3} \pm 0.56\cdot 10^{-3}\ s^{-1}$. *Source data are provided as a Source Data file.*

Supplementary Note 6. Pyruvate to pyruvate hydrate ratio as a function of temperature

The temperature dependence of the pyruvate-to-pyruvate hydrate (Py/PyH) ratio is examined to investigate the dynamic exchange between oxo (Py) and hydrated (PyH) forms. Temperature influences both the equilibrium position and the exchange rate. By analyzing this dependence, we aim to understand how temperature affects the thermodynamics of pyruvate exchange in aqueous solution.

Sample: sample #I, [pyruvate] = 90.94 mM in 90% H_2O and 10% D_2O.

NMR pulse sequences:

^{1}H 90°-FID (free induction decay) sequence with the following parameters was used d1=30 s (relaxation delay), aq = 3.99 s (acquisition time), sw = 20.48 ppm.

^{13}C 90°-FID sequence with proton decoupling during FID was used with the following parameters: d1 = 200 s, aq=3.99 s, sw=81.4541 ppm.

Method: ^{1}H and ^{13}C spectra were acquired for sample #I with the two sequences described above. Spectra were acquired on a Bruker Avance NEO WB400 NMR spectrometer. Data acquisition and instrument control were performed using Bruker Topspin 4.1.4 software. The spectrometer was locked to D_2O during all measurements. Experiments were carried out at 283, 293, 303, 313, 323, 333 K. Data processing and analysis were performed using MNova and OriginPro 2021b.

Results: exemplary ^{1}H and ^{13}C NMR spectra are presented in Fig. S7. In both cases Py and PyH lines are well resolved.

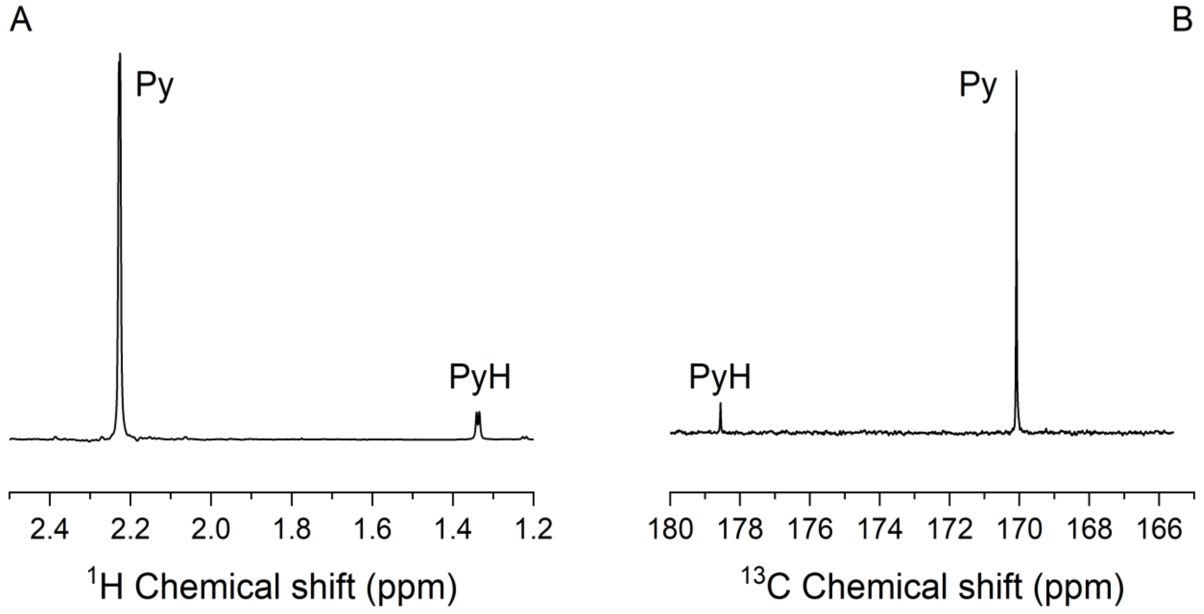

Fig. S7. *^{1}H (a) and ^{13}C (b) NMR spectra of sample #I at 283 K. ^{1}H chemical shift difference is 0.9 ppm, ^{13}C chemical shift difference is 8.6 ppm. Source data are provided as a Source Data file.*

Tab. S6. *[Py]/[PyH] ratio as a function of temperature. The ratio fits well with the Arrhenius equation.*

T (K)	Exp ID	[Pyr]/[PyrOH]
283	20	7.9
293	10	10.37
303	30	14.31
313	70	18
323	50	23.77
333	60	28.59

Supplementary Note 7. Chemical exchange rate between pyruvate and pyruvate hydrate

Here, we discuss how we estimated the exchange rates between Py and PyH.

Sample: sample #I, [pyruvate] = 90.94 mM in 90% H_2O and 10% D_2O

NMR pulse sequence: ^{13}C SWAP-IR sequence with proton decoupling during FID was used with the following parameters: d1 = 30 s, aq=3 s, sw=81.4541 ppm, o1p = 170.08 ppm, o2p = 2.23 ppm.

The SWAP-IR sequence Linden et al.[11] with refocusing was used without changes and without phase cycling:

^{13}C:-90_X-τ-180_X-τ-$90_X 90_Y$-τ-180_X-τ-90_{-Y}-(1 ms, PFG SMSQ10.100, 11%)-vd-90_X-FID

^{1}H:-90_X-τ-180_X-τ-$90_X 90_Y$-τ-180_X-τ-90_Y---decoupling

The SWAP is almost identical to the INEPT with refocusing but should provide distortion-free lines since it does not populate multiplet spin order. As a result of this excitation, Py signals are polarized and then by varying vd list we could probe the exchange between Py and PyH.

The following vd list was used: 0, 1, 2, 3, 4, 5, 6, 8, 10, 12, 14, 16, 18, 20, 24, 28, 32, 48, 64, 96, 128, 160, 192, 256, 384 s with τ = 90 ms.

Method: When all experiments were conducted, the spectra were analysed using MNova and OriginPro 2021b software. Py and PyH lines were integrated and the integral were fitted with a bi-exponential decay function, $A_1\exp(-k \cdot t) + A_2\exp(-R \cdot t) + y_0$ where the y_0 represent the offset, A_1 and A_2 are the amplitude and k and R are the eigen values of Bloch-Macconnel equation with chemical exchange (Eq. S1). When the exchange is much faster than relaxation, then one can extract the exchange rates from k and ratio of concentrations Salnikov et al[12]:

$$\begin{cases} k \cong (k_1 + k_{-1}) = k_{-1}\left(1 + \frac{k_1}{k_{-1}}\right) = k_{-1}(1 + K_{\mathrm{eq}}) \\ K_{\mathrm{eq}} = \frac{k_1}{k_{-1}} = \frac{[\mathrm{PyH}]}{[\mathrm{Py}]} \\ k_{-1} \cong \frac{k}{1 + K_{\mathrm{eq}}} = \frac{k}{1 + \frac{[\mathrm{PyH}]}{[\mathrm{Py}]}} \\ k_1 = k_{-1}K_{\mathrm{eq}} \end{cases} \quad \textbf{Eq. S4}$$

Hence (Tab. S7), indeed exchange is much faster than relaxation.

***Tab. S7**. Estimated exchange rate constants from ^{13}C SWAP-IR experiment.*

T (K)	Exp ID for K_{eq}	[Pyr]/ [PyrOH]	$K_{\mathrm{eq}} = \frac{k_1}{k_{-1}} = [\mathrm{PyH}]/[\mathrm{Py}]$	Exp ID for k	R s^{-1}	k s^{-1}	k_{-1} s^{-1}	k_1 s^{-1}
283	20	7.9	0.13	22	0.027±0.0006	0.095±0.024	0.085±0.02	0.01±0.003
293	10	10.37	0.096	12	0.025±0.0005	0.19±0.065	0.174±0.06	0.017±0.006
303	30	14.31	0.07	32	0.021±0.0003	0.233±0.07	0.22±0.06	0.015±0.005

Supplementary References

1. Frisch, M. J.; Trucks, G. W.; Schlegel, H. B.; Scuseria, G. E.; Robb, M. A.; Cheeseman, J. R.; Scalmani, G.; Barone, V.; Petersson, G. A.; Nakatsuji, H.; Li, X.; Caricato, M.; Marenich, A. V.; Bloino, J.; Janesko, B. G.; Gomperts, R.; Mennucci, B.; Hratchian, H. P.; Ortiz, J. V.; Izmaylov, A. F.; Sonnenberg, J. L.; Williams-Young, D.; Ding, F.; Lipparini, F.; Egidi, F.; Goings, J.; Peng, B.; Petrone, A.; Henderson, T.; Ranasinghe, D.; Zakrzewski, V. G.; Gao, J.; Rega, N.; Zheng, G.; Liang, W.; Hada, M.; Ehara, M.; Toyota, K.; Fukuda, R.; Hasegawa, J.; Ishida, M.; Nakajima, T.; Honda, Y.; Kitao, O.; Nakai, H.; Vreven, T.; Throssell, K.; Montgomery, J. A., Jr.; Peralta, J. E.; Ogliaro, F.; Bearpark, M. J.; Heyd, J. J.; Brothers, E. N.; Kudin, K. N.; Staroverov, V. N.; Keith, T. A.; Kobayashi, R.; Normand, J.; Raghavachari, K.; Rendell, A. P.; Burant, J. C.; Iyengar, S. S.; Tomasi, J.; Cossi, M.; Millam, J. M.; Klene, M.; Adamo, C.; Cammi, R.; Ochterski, J. W.; Martin, R. L.; Morokuma, K.; Farkas, O.; Foresman, J. B.; Fox, D. J. Gaussian 16 Revision C.01, 2016. Gaussian 16 Revision A.03.
2. Abraham, M. *et al.* GROMACS 2023.2 Manual. https://doi.org/10.5281/ZENODO.8134388 (2023) doi:10.5281/ZENODO.8134388.
3. Vanommeslaeghe, K. *et al.* CHARMM general force field: A force field for drug-like molecules compatible with the CHARMM all-atom additive biological force fields. *J. Comput. Chem.* **31**, 671–690 (2010).
4. Kowalewski, J. & Maler, L. *Nuclear Spin Relaxation in Liquids: Theory, Experiments, and Applications*. (CRC Press, 2006). doi:10.1201/9781420012194.
5. Hwang, L.-P. & Freed, J. H. Dynamic effects of pair correlation functions on spin relaxation by translational diffusion in liquids. *J. Chem. Phys.* **63**, 4017–4025 (1975).
6. Teng, C.-L., Hong, H., Kiihne, S. & Bryant, R. G. Molecular Oxygen Spin–Lattice Relaxation in Solutions Measured by Proton Magnetic Relaxation Dispersion. *J. Magn. Reson.* **148**, 31–34 (2001).

7. Livo, K., Prasad, M. & Graham, T. R. Quantification of dissolved O2 in bulk aqueous solutions and porous media using NMR relaxometry. *Sci. Rep.* **11**, 290 (2021).
8. Bondi, A. van der Waals Volumes and Radii. *J. Phys. Chem.* **68**, 441–451 (1964).
9. Jamnongwong, M., Loubiere, K., Dietrich, N. & Hébrard, G. Experimental study of oxygen diffusion coefficients in clean water containing salt, glucose or surfactant: Consequences on the liquid-side mass transfer coefficients. *Chem. Eng. J.* **165**, 758–768 (2010).
10. Koelsch, B. L. *et al.* Diffusion MR of hyperpolarized 13C molecules in solution. *Analyst* **138**, 1011 (2013).
11. Linden, N., Barjat, H., Kupče, Ē. & Freeman, R. How to exchange information between two coupled nuclear spins: the universal SWAP operation. *Chem. Phys. Lett.* **307**, 198–204 (1999).
12. Salnikov, O. G. *et al.* Modeling Ligand Exchange Kinetics in Iridium Complexes Catalyzing SABRE Nuclear Spin Hyperpolarization. *Anal. Chem.* **96**, 11790–11799 (2024).
