# Supplementary material for: Over four minutes of pyruvate T1 using chemically and physically induced deceleration of relaxation
Source: Nat Commun. 2026 May 21;17:4561. doi: 10.1038/s41467-026-73214-w (PMC13194983; doi:10.1038/s41467-026-73214-w)
Supplement: Supplementary file 2 — Reporting Summary [file 41467_2026_73214_MOESM2_ESM.pdf]

nature portfolio

Corresponding author(s): Andrey N. Pravdivtsev, Josh P. Peters

Last updated by author(s): Mar 3, 2026

Reporting Summary

Nature Portfolio wishes to improve the reproducibility of the work that we publish. This form provides structure for consistency and transparency in reporting. For further information on Nature Portfolio policies, see our Editorial Policies and the Editorial Policy Checklist.

Statistics

For all statistical analyses, confirm that the following items are present in the figure legend, table legend, main text, or Methods section.

n/a	Confirmed	
☐	☒	The exact sample size (n) for each experimental group/condition, given as a discrete number and unit of measurement
☐	☒	A statement on whether measurements were taken from distinct samples or whether the same sample was measured repeatedly
☒	☐	The statistical test(s) used AND whether they are one- or two-sided<br>*Only common tests should be described solely by name; describe more complex techniques in the Methods section.*
☒	☐	A description of all covariates tested
☒	☐	A description of any assumptions or corrections, such as tests of normality and adjustment for multiple comparisons
☒	☐	A full description of the statistical parameters including central tendency (e.g. means) or other basic estimates (e.g. regression coefficient) AND variation (e.g. standard deviation) or associated estimates of uncertainty (e.g. confidence intervals)
☒	☐	For null hypothesis testing, the test statistic (e.g. F, t, r) with confidence intervals, effect sizes, degrees of freedom and P value noted<br>*Give P values as exact values whenever suitable.*
☒	☐	For Bayesian analysis, information on the choice of priors and Markov chain Monte Carlo settings
☒	☐	For hierarchical and complex designs, identification of the appropriate level for tests and full reporting of outcomes
☒	☐	Estimates of effect sizes (e.g. Cohen's d, Pearson's r), indicating how they were calculated

Our web collection on statistics for biologists contains articles on many of the points above.

Software and code

Policy information about availability of computer code

Data collection	TopSpin 4.4.0 (Bruker), SpinsolveExpert 2.02.00 (Magritek), magnetic-field-cycling hardware and software described in doi: 10.1021/acsmeasuresciau.5c00131. GROMACS 2023.3, Gaussian 16 ES64L-G16RevA.03, GROMACS 2023.3, and ORCA 5.0.2 software were used for computational chemistry simulations.
Data analysis	Mestrenova 15.1.0-38027 (Mestrelab Research S.L.), OriginPro 9.8.5.201 2021b (OriginLab Corporation), and Mathematica 14.0.0.0

For manuscripts utilizing custom algorithms or software that are central to the research but not yet described in published literature, software must be made available to editors and reviewers. We strongly encourage code deposition in a community repository (e.g. GitHub). See the Nature Portfolio guidelines for submitting code & software for further information.

Data

Policy information about availability of data

All manuscripts must include a data availability statement. This statement should provide the following information, where applicable:

- Accession codes, unique identifiers, or web links for publicly available datasets
- A description of any restrictions on data availability
- For clinical datasets or third party data, please ensure that the statement adheres to our policy

Raw data, MD input parameters, DFT calculated geometries, and Mathematica scripts can be accessed via Zenodo DOI: https://doi.org/10.5281/zenodo.17175125.

Research involving human participants, their data, or biological material

Policy information about studies with human participants or human data. See also policy information about sex, gender (identity/presentation), and sexual orientation and race, ethnicity and racism.

Reporting on sex and gender	n.a.
Reporting on race, ethnicity, or other socially relevant groupings	n.a.
Population characteristics	n.a.
Recruitment	n.a.
Ethics oversight	n.a.

Note that full information on the approval of the study protocol must also be provided in the manuscript.

Field-specific reporting

Please select the one below that is the best fit for your research. If you are not sure, read the appropriate sections before making your selection.

- [x] Life sciences
- [ ] Behavioural & social sciences
- [ ] Ecological, evolutionary & environmental sciences

For a reference copy of the document with all sections, see nature.com/documents/nr-reporting-summary-flat.pdf

Life sciences study design

All studies must disclose on these points even when the disclosure is negative.

Sample size	The sample size for statistical test was N=2 for D2O filtr. and N=3 for D2O and H2O cell experiments in Fig. 4. Repetitions were performed using biological repeats and independent from one another. 3 biological repeats were aspired to for statistical analysis, no sample size calculation was performed. Strong differences between the groups were expected and dDNP experiments take several hours each, so sample size was kept at a minimum.
Data exclusions	One sample from D2O filtr. was excluded, since the cells were grown in a T175 instead of T75 flask like the other biological repeats.
Replication	All attempts at replication were successfull, as long as DNP dissolution did not fail.
Randomization	We performed measurements across 3 days and tried to group D2O filtr., D2O, and H2O together on each days to achieve technical reproducibility. On the last day, D2O and H2O were both measured. As the alteration to the dissolution medium was the only planned experimental variable, covariates are expected to be the same for each experiment.
Blinding	Investigators were not blinded, as the specific dissolution medium had to be prepared and loaded before performing the specific experiment. The blinding is not expected to alter the observed and fitted T1 and conversion.

Reporting for specific materials, systems and methods

We require information from authors about some types of materials, experimental systems and methods used in many studies. Here, indicate whether each material, system or method listed is relevant to your study. If you are not sure if a list item applies to your research, read the appropriate section before selecting a response.

Materials & experimental systems

n/a	Involved in the study	
x		Antibodies
	x	Eukaryotic cell lines
x		Palaeontology and archaeology
x		Animals and other organisms
x		Clinical data
x		Dual use research of concern
x		Plants

Methods

n/a	Involved in the study	
x		ChIP-seq
x		Flow cytometry
x		MRI-based neuroimaging

Eukaryotic cell lines

Policy information about cell lines and Sex and Gender in Research

Cell line source(s)	Human cervical cancer HeLa cells from ATCC: CCL-2, thawed from frozen stock and seeded for the experiments. Sex: female.
Authentication	None of the cell lines used were authenticated, though the specific cell line is not per se relevant as long as it is the same among the different groups.
Mycoplasma contamination	MycoAlert assay (from Lonza), none were found in 2012.
Commonly misidentified lines (See ICLAC register)	none

Plants

Seed stocks	*Report on the source of all seed stocks or other plant material used. If applicable, state the seed stock centre and catalogue number. If plant specimens were collected from the field, describe the collection location, date and sampling procedures.*
Novel plant genotypes	*Describe the methods by which all novel plant genotypes were produced. This includes those generated by transgenic approaches, gene editing, chemical/radiation-based mutagenesis and hybridization. For transgenic lines, describe the transformation method, the number of independent lines analyzed and the generation upon which experiments were performed. For gene-edited lines, describe the editor used, the endogenous sequence targeted for editing, the targeting guide RNA sequence (if applicable) and how the editor was applied.*
Authentication	*Describe any authentication procedures for each seed stock used or novel genotype generated. Describe any experiments used to assess the effect of a mutation and, where applicable, how potential secondary effects (e.g. second site T-DNA insertions, mosiacism, off-target gene editing) were examined.*
